# The Influence of Sulfation Degree of Glycosaminoglycan-Functionalized 3D Collagen I Networks on Cytokine Profiles of In Vitro Macrophage–Fibroblast Cocultures

**DOI:** 10.3390/gels10070450

**Published:** 2024-07-09

**Authors:** Franziska Ullm, Alexander Renner, Uwe Freudenberg, Carsten Werner, Tilo Pompe

**Affiliations:** 1 Institute of Biochemistry, Leipzig University, Johannisallee 21-23, 04103 Leipzig, Germany; franziska.ullm@gmx.de (F.U.); alexander.renner@izi.fraunhofer.de (A.R.); 2 Max Bergmann Center of Biomaterials, Leibniz-Institut für Polymerforschung Dresden e.V., Hohe Strasse 6, 01069 Dresden, Germany; freudenberg@ipfdd.de (U.F.); werner@ipfdd.de (C.W.)

**Keywords:** 3D hydrogels, collagen I networks, glycosaminoglycans, coculture, macrophages, wound healing

## Abstract

Cell–cell interactions between fibroblasts and immune cells, like macrophages, are influenced by interaction with the surrounding extracellular matrix during wound healing. In vitro hydrogel models that mimic and modulate these interactions, especially of soluble mediators like cytokines, may allow for a more detailed investigation of immunomodulatory processes. In the present study, a biomimetic extracellular matrix model based on fibrillar 3D collagen I networks with a functionalization with heparin or 6-ON-desulfated heparin, as mimics of naturally occurring heparan sulfate, was developed to modulate cytokine binding effects with the hydrogel matrix. The constitution and microstructure of the collagen I network were found to be stable throughout the 7-day culture period. A coculture study of primary human fibroblasts/myofibroblasts and M-CSF-stimulated macrophages was used to show its applicability to simulate processes of progressed wound healing. The quantification of secreted cytokines (IL-8, IL-10, IL-6, FGF-2) in the cell culture supernatant demonstrated the differential impact of glycosaminoglycan functionalization of the collagen I network. Most prominently, IL-6 and FGF-2 were shown to be regulated by the cell culture condition and network constitution, indicating changes in paracrine and autocrine cell–cell communication of the fibroblast–macrophage coculture. From this perspective, we consider our newly established in vitro hydrogel model suitable for mechanistic coculture analyses of primary human cells to unravel the role of extracellular matrix factors in key events of tissue regeneration and beyond.

## 1. Introduction

Upon injury, epithelial, endothelial and blood cells release inflammatory mediators that initiate an antifibrinolytic coagulation cascade, which triggers platelet aggregation, clot formation, and the development of a provisional extracellular matrix (ECM). The subsequent process of wound healing is highly complex and interdigitated and requires the coordinated interaction of different cell types during the four main phases of wound healing, namely hemostasis, inflammation, proliferation, and remodeling [1,2]. In all these phases, the surrounding ECM plays a crucial function by modulating cell-ECM adhesion and binding soluble mediators like cytokines through its highly hydrated biopolymer network, consisting of fibrillar protein networks and glycosaminoglycan decorations.

Two cell types that are critically involved in the progression of functional wound healing are macrophages (MΦ) and fibroblasts (Fb). MΦ either originate from circulating blood monocytes or persist in tissues as part of the immune system. Their multifunctionality during homeostasis, tissue repair, and the restoration of tissue integrity can be attributed to their broad spectrum of released cytokines and growth factors, which also makes them important players in mediating immune responses [3,4]. There are different pathways of MΦ activation, leading to different subtypes with specific secretion profiles and functions but also to different interaction possibilities with the surrounding ECM and cells in the context of wound healing. Furthermore, as fully differentiated cells, MΦ still possess high phenotypic plasticity in response to changing environmental conditions, both in vivo and in vitro [5]. During nearly all wound healing phases, a closed physical proximity of MΦ and Fb occurs, allowing for direct cell–cell interaction via soluble mediators over short distances. In this context, MΦ subtypes are known to affect Fb differentiation in diverging ways due to distinct secretion profiles [6,7,8,9]. After reaching the wound site, Fb proliferate and differentiate into myofibroblasts (MyoFb), mainly in response to fibroblast growth factor 2 (FGF-2), transforming growth factor β_1_ (TGF-β_1_), and an increased stiffness of the surrounding tissue. They are mainly characterized by the incorporation of alpha smooth muscle actin (αSMA), facilitate wound closure by contractile forces, and begin to replace the provisional ECM by producing large amounts of collagen (Coll) type I and III [10,11].

Although the physiological process of cutaneous wound healing and the underlying mechanisms are known and can be controlled in the clinic, pathologic situations, such as chronic inflammatory wounds, excessive scarring, or fibrosis, have not been adequately understood, in part due to the lack of appropriate investigative tools and model systems. Particularly, the regulation of the interaction between MΦ and Fb plays a crucial role in such pathological situations, which is also reflected in a large number of recent reports [12,13,14,15,16].

To study complex healing processes like de- or transdifferentiation of human MyoFb in the presence of polarized or polarizing MΦ, suitable in vitro models using Fb-MΦ cocultivation are needed. Those model systems should closely mimic the 3D ECM context at different phases of wound healing as well as allowing for the application of human and primary cell models and access for methods with high temporal and local resolution [17]. In particular, successful biomimetic coculture systems should allow for the interaction of cells within a rebuilt and nearly native 3D ECM. The composition and microstructure of the ECM play a crucial role as they form the temporary scaffold during in vivo wound healing and ensure the structural integrity of the wound model during the entire course of study [18,19]. Three-dimensional fibrillar networks exhibit many key properties of the natural ECM, such as low stiffness, micron-sized pores, and fibrillar microstructure, constituting a highly functional hydrogel matrix. Therefore, they are promising candidates for accurately mimicking the ECM microenvironment of cells [20]. Since Coll I is the most abundant ECM protein in vivo and well present in the cutaneous ECM, Coll-based scaffolds are very suitable for cell studies and are considered the gold standard. In vitro reconstituted 3D fibrillar Coll I networks were shown to enable cell migration and invasion, allow for matrix degradation via matrix metalloproteases (MMPs), and exhibit specific cell receptor binding sites as well as native-like viscoelastic and tunable mechanical properties [21,22,23,24].

Coll I-based networks can be further modified with other components of the natural ECM, including glycosaminoglycans (GAGs) (e.g., heparin) [25,26], proteoglycans (e.g., aggrecan), or fibronectin [27] to adapt for an even more precise mimic of the natural ECM. However, such modifications with tight mimics of naturally occurring GAGs like heparan sulfate and their application in advanced coculture studies are still missing. GAGs are of central importance due to their capability to bind a variety of signaling molecules like cytokines and growth factors [28]. GAGs consist of repetitive disaccharide subunits, with iduronic or glucuronic acid and N-acetyl-glucosamine being the most abundant building blocks of the naturally occurring heparan sulfates in the ECM. Depending on the tissue, differences exist in the degree and pattern of sulfation, which are most important for cytokine binding [29,30]. In that way, the binding of soluble mediators, adjustment of matrix mechanical properties as well as intercellular, cell–matrix, and cell–cell interactions can be modulated. For controlling wound healing processes in the context of medical applications to treat acute and chronic wounds, the binding and release of cytokines and growth factors were shown to be of advantage, affecting the immunomodulatory responses [31,32]. Heparin, as one type of heparan sulfate, is frequently used as a broadly available highly sulfated GAG with high-affinity binding characteristics towards many signaling molecules [33,34]. Heparin has already been shown to have a beneficial influence on patient wound healing as a supplemental component of artificial ECM [35]. However, native heparan sulfate GAGs of the ECM exhibit only one sulfate group per disaccharide unit instead of three sulfates per disaccharide for heparin. As native heparan sulfate is hardly available in reasonable amounts and reproducible batch quality for the biomimetic matrix engineering of 3D scaffolds, a selectively 6-ON-desulfated variant of heparin was recently introduced and shown to allow for a selective adjustment of binding and release of various cytokines [36,37].

Our new study aims to implement and investigate the modulating function of tight mimics of naturally occurring heparan sulfate GAGs in a recently established 3D coculture model of primary human Fb and MΦ [38]. By using direct coculture models with primary human cells as opposed to established cell lines, intercellular communication during wound healing can be elucidated much closer to the biomedical situation. The successful implementation of close mimics of fibrillar and heparan sulfate-modified ECM and primary human coculture is intended to reduce the need for animal models in biomedical research [39]. In our approach, we focused on a representation of a late-stage wound healing scenario, where primary human (Myo)Fb and M-CSF-polarized MΦ (M-MΦ) interact within a fibrillar 3D Coll I-based hydrogel network with a modulation of cell–cell interaction by matrix-bound heparin or 6-ON-desulfated heparin.

## 2. Results and Discussion

### 2.1. Enabling Sulfated Glycosaminoglycan Modification of a 3D Collagen-Based Biomimetic Coculture Model

To achieve a more appropriate representation of the ECM microenvironment at late stages of wound healing, we aimed to modify our previously established biomimetic 3D Coll I coculture model by coupling sulfated GAGs to the fibrillar microstructure of the 3D Coll I matrix [38]. Therefore, successful binding of heparin and 6-ON-desulfated heparin to the Coll I matrix had to be ensured. Due to their differential binding capacities for various soluble mediators such as cytokines and growth factors, both GAGs should enable the targeted modulation of cellular interactions to a different degree [37]. As a first step, the following points needed to be investigated to ensure stable experimental conditions: Coll I fibrillation process, adsorptive heparin coupling to the matrix (amount and stability), and cell viability after matrix reconstitution and GAG coupling.

### 2.2. Coll I Fibril Formation Is Unaltered at Cell Presence

**Figure 1:**
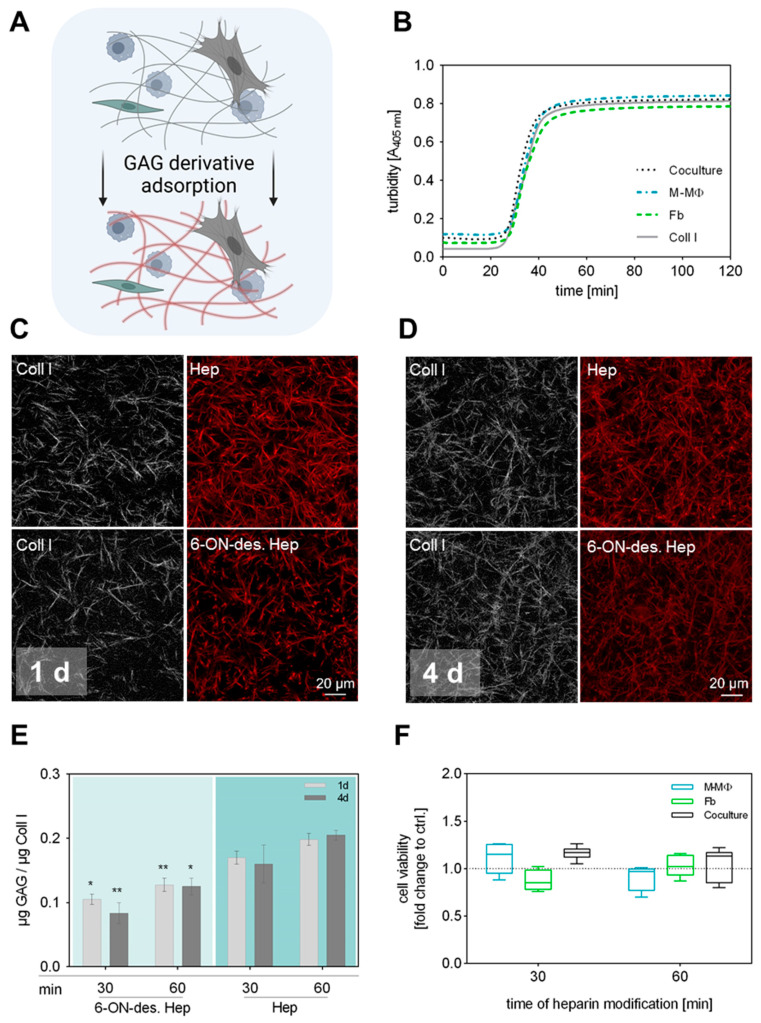
Preparation and characterization of 3D GAG-modified Coll I matrices. (**A**) Schematic illustration of performed GAG modification of Coll I matrices. To maintain the 3D distribution of both cell types in the network, GAG solutions were applied after Coll I fibrillation was completed. Figure was generated using biorender.com (accessed on 11 June 2024). (**B**) Turbidity measurement of Coll I fibrillogenesis performed over 120 min at 405 nm with and without cells present in the Coll I solution. (**C**,**D**) Representative cLSM images of Coll I matrices showing atto550-labelled variants of heparin and 6-ON-desulfated heparin (red) either 1 d (**C**) or 4 d (**D**) after reconstitution. Scale bar 20 µm. (**E**) Analysis of GAG amount in Coll I matrices 1 d and 4 d after reconstitution using papain digestion (*n* = 6; data is given as mean ± SE; Tukey’s multiple comparisons test followed by two-way ANOVA; * indicates *p* ≤ 0.05; ** indicates *p* ≤ 0.005 between the respective heparin variants; no significant differences between 1 d and 4 d comparison). (**F**) Analysis of cell viability after 4 d of cell culture exemplarily shown for heparin-modified Coll I matrices after 30 and 60 min of GAG incubation (*n* = 3, data is not significant after ordinary one-way ANOVA, Box-whisker plots show the 25 and 75 percentile range (box) with Tukey 95% confidence intervals (whiskers) and median values (transversal line); dotted line represents respective Coll I control without GAG modification).

Typically, our sequential protocols for engineering functionalized Coll I matrices with defined microstructure and modification involve two steps: (1) Coll I fibrillation followed by (2) adsorptive or covalent binding of other ECM components such as fibronectin or GAGs [25,27,40]. In our previously established 3D coculture model of Fb and MΦ, the first step is extended by the incorporation of both cell types already during Coll I fibrillation to achieve a homogeneous 3D distribution within the matrix [38]. Accordingly, the conditions of the subsequent GAG modification had to be adjusted in a way that the introduced cells were not adversely affected. The sequential procedure and experimental setup are schematically shown in Figure 1A. Since it is well known that the kinetic of fibrillation significantly determines the resulting microstructure of the Coll I network (see Figure 1C,D) [41], we first checked whether there are differences during fibril formation between Coll I solutions with and without incorporated cells (Figure 1B). Turbidity measurements at 405 nm over 120 min showed that fibril formation had the same kinetics in all cell conditions, namely with incorporated Fb, M-MΦ, and both cell types, also in comparison to controls without cells. In general, it was observed that fibril formation terminated after 50 min. In conclusion, the presence of cells did not interfere with the kinetics of fibril formation of Coll I in this approach.

### 2.3. Stable Coupling of Heparin and 6-ON-Desulfated Heparin to Coll I Matrices

Due to the cell presence in the reconstituted Coll I matrices, covalent binding of GAGs like heparin using carbodiimide chemistry, as previously established, was not applicable in the current setting. However, previous studies indicated that sulfated GAGs can be adsorptively coupled to Coll I matrices with high affinity and stability [25]. In order to investigate the adsorptive coupling of heparin and 6-ON-desulfated heparin to reconstituted Coll I matrices, we used fluorescently labelled GAGs and checked their presence via confocal laser scanning microscopy (cLSM) and fluorimetry. Coll I matrices were incubated with solutions of 0.1 mg mL^−1^ of atto550-heparin or atto550-6-ON-desulfated heparin in PBS for either 30 or 60 min, followed by incubation at standard cell culture conditions for 1 d and 4 d and subsequent quantification of the GAG amount in the matrices. The results showed a homogenous and stable binding of both heparin variants to the fibrillar microstructure of the Coll I matrix. Approx. 0.1 μg 6-ON-desulfated heparin and 0.15 μg heparin per μg Coll I were found to be present in the matrix after a duration of 4 d (Figure 1C–E). The adsorptive amount of both heparin variants was found to be slightly, but not significantly, higher for a 60 min incubation time, instead of 30 min. Data on heparin coupling to Coll I matrices without incorporated cells are additionally shown in Supplementary Figure S1.

The difference in the adsorbed amount of heparin and 6-ON-desulfated heparin has to be attributed to the degree of sulfation of these molecules. Since 6-ON-desulfated heparin has only 34% of sulfate groups in comparison to heparin, the overall negative charge of 6-ON-desulfated heparin is decreased at the used cell culture conditions [36], leading to less adsorption to Coll I fibrils, as also shown in our earlier studies [25]. Nevertheless, 6-ON-desulfated heparin was adsorbed in a stable manner to Coll I matrices at cell culture conditions over 4 days. In conclusion, heparin and 6-ON-desulfated heparin can be homogenously and stably adsorbed to 3D Coll I matrices in similar amounts, which provides the basis for a 3D fibrillar Coll I-based matrix model with modification by sulfated GAGs to modulate cellular interaction.

### 2.4. Cell Viability Is Not Affected by GAG Modification of Coll I Matrices

Prior to the coculture study, we checked whether a GAG modification of Coll I matrices stresses seeded cells with detrimental effects on cell viability. Cell viability in modified 3D Coll I matrices was determined by WST-1 assay after 4 d of cell culture for GAG incubation times of 15 min, 30 min, or 60 min (Supplementary Figure S2). Unmodified Coll I matrices were used as controls. Cell viability was not significantly altered when the heparin solution was applied for 30 and 60 min to cell-containing scaffolds, respectively (Figure 1F). Viability in coculture samples was slightly, but not significantly, higher than for reference samples. This effect can be attributed to an increase in cell number after 4 d of cultivation.

Combining the results of GAG adsorption and cell viability, a final modification time of Coll I matrices with heparin solutions of 30 min was chosen to be most suitable for all follow-up experiments. The adsorptive amounts of heparin and 6-ON-desulfated heparin were only non-significantly increased after 60 min of incubation. Furthermore, a 30 min GAG incubation minimizes detrimental conditions for the cells during Coll I matrix reconstitution and modification.

### 2.5. Stable Microstructure of GAG-Modified Coll I Matrices during Cell Culture

To ensure stable cell culture conditions within the scaffold, we examined the matrix microstructure using our established tools for topology analysis of 3D fibrillar Coll I matrices. The analysis revealed no changes in mean pore diameter, fibril diameter, and fibril density for all scaffold types and cell culture conditions (Supplementary Figure S3). This suggests that the used Coll I matrix provides a stable and reproducible scaffold for the cells throughout the culture period of 7 days, with a similar matrix microstructure. This finding agrees with our previous report on 3D fibrillar Coll I matrices without a GAG modification, showing similar network topology and layer thickness of the 3D construct over 7 days [38]. However, one should keep in mind that the structural stability of fibrillar Coll I matrices depends strongly on the used cell type, as previous studies using human mesenchymal stromal cells showed strong degradation of non-crosslinked fibrillar Coll I matrices by matrix metalloproteinases [42].

In sum, a 3D Coll I-based biomimetic model was set up by coupling differently sulfated GAGs, namely heparin and 6-ON-desulfated heparin, to 3D fibrillar Coll I matrices for stable constitution and microstructure for cell culture studies.

### 2.6. Differential Cytokine Production by GAG-Mediated Cell–Cell Signaling

**Figure 2:**
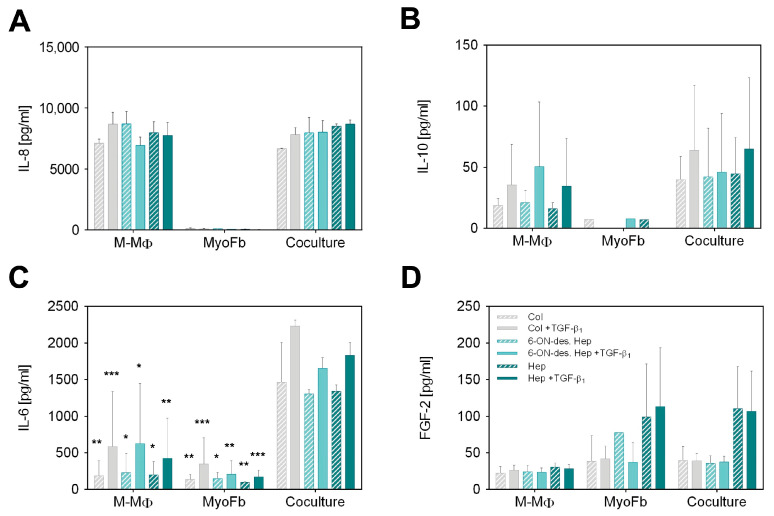
Cytokine secretion after 7 d in GAG-modified Coll I matrices. Secretion of (**A**) IL-8, (**B**) IL-10, (**C**) IL-6 and (**D**) FGF-2 by M-MΦ, MyoFb and in coculture. Concentration of cytokines (pg mL^−1^) in supernatants was determined by multiplex assay after 7 d using a standard curve. Data are shown as mean ± SD from 3 separate cell experiments. Levels of significance are shown against the respective coculture condition and were set to *p* ≤ 0.05 (*) using Tukey’s multiple comparisons test followed by two-way ANOVA, whereas *p* ≤ 0.01 (**) and *p* ≤ 0.001 (***) represents very and highly significant, respectively.

To determine whether the binding of heparin or 6-ON-desulfated heparin affects cellular communication between MyoFb and M-MΦ, we examined both mono- and cocultures after 7 days using a multiplex ELISA assay. Figure 2 shows the levels of four relevant cytokines in the culture supernatant (IL-8, IL-10, IL-6, FGF-2), which are known to be involved in Fb/MyoFb and MΦ cell–cell signaling in wound healing but exhibit quite different characteristics in terms of cellular origin and interaction with sulfated GAGs [33,37]. From the cytokine profile of the different scaffolds and culture conditions, we found different interaction mechanisms of cytokines with the GAG-modified matrix and a differential impact on cell–cell communication.

Analysis of IL-8 as a classical pro-inflammatory cytokine shown in Figure 2A revealed very high levels in M-MΦ monocultures and cocultures, whereas MyoFb did not produce IL-8, as expected. In agreement, there was no change in IL-8 levels by TGF-β_1_ stimulation, as TGF-β_1_ is known to primarily act on Fb. No dependency on the specific GAG modification of the Coll I matrix is apparent, too. Although IL-8, as a highly negatively charged cytokine with an isoelectric point (IEP) of 9.2, is known to strongly bind to heparin but also to desulfated heparin [37], we did not observe different IL-8 levels in the supernatant resulting from any heparin modifications of the matrix. From this result, one can conclude that the amount of heparin present in our scaffold system is too low to affect the high IL-8 levels in the supernatant (approx. 8000 pg mL^−1^). This conclusion is supported by comparing the GAG amounts present in our setup in comparison to a previous study on GAG-mediated IL-8 sequestering by starPEG-heparin hydrogels [31,37]. Here, approximately 50 µg GAG per µL hydrogel volume was present in comparison to 0.25 µg per µL matrix volume in our fibrillar Coll I matrix, being two orders of magnitude lower.

Next, we analyzed IL-10, as IL-10 expressed by MΦ plays a crucial role, specifically in the remodeling phase of late wound healing. IL-10 supports the de-differentiation of MyoFb and, thus, significantly stops the progressive formation of a new matrix. The results presented in Figure 2B show an increase in IL-10 production in response to TGF-β_1_ in M-MΦ monocultures, which was similarly observed in the coculture setting but at slightly higher levels. Again, no IL-10 was detected in the MyoFb monoculture, as expected, because M-MΦ are known as primary IL-10 secreting cells. The TGF-β_1_-dependent activation of M-MΦ mono- and cocultures was previously not observed in our setup [38] but is reasonable due to the activation of anti-inflammatory pathways in M-MΦ by the presence of MyoFb, resulting in an increased production of IL-10 [43]. The stronger data scatter has to be attributed to the well-known donor dependence of TGF-β_1_ activation of primary Fb and the interaction of MyoFb and M-MΦ, as discussed above. Similarly to IL-8, we have to state no difference in IL-10 levels with respect to the heparin modification of the Coll I matrix. IL-10 was found at much lower levels than Il-8 (two orders of magnitude lower). Hence, the above argument for the limited influence of the relatively low amount of GAG in the matrix might be not valid, as for IL-8. However, for IL-10, another crucial factor comes into play. IL-10 is known to weakly bind to sulfated GAGs only [37]. Therefore, the sulfation degree of heparin and 6-ON-desulfated heparin cannot be expected to additionally modulate the levels of IL-10 in cell culture media triggered by the different cell culture conditions.

We further analyzed the weakly charged cytokine IL-6 with an IEP of 6.2. It has anti-apoptotic properties and regulates the polarization of anti-inflammatory MΦ in vivo in an autocrine and paracrine manner. In our setup, both cell types produce IL-6 (Figure 2C). Regardless of the matrix GAG modification, the measured levels were doubled by TGF-β_1_ activation for M-MΦ as well as for MyoFb. Compared to the monocultures, a nearly 10-fold increase in IL-6 was found in the supernatant of cocultures up to a range of 1500 pg mL^−1^. Interestingly, the large scatter between individual donors was strongly decreased in the coculture. These results indicate that the coculture of MyoFb and M-MΦ increases the paracrine action of IL-6 and leads to a feedback loop, “synchronizing” IL-6 levels in the supernatant. Similar results were recently shown in another study, where the amount of IL-6 produced by the Fb monoculture was significantly increased using a conditioned medium from CD206^+^-MΦ [15]. Since MyoFb and M-MΦ are capable of producing IL-6 [44], a mutual interference is quite conceivable. IL-6 from MΦ has been shown to reduce the gene expression of Coll type I and III as well as αSMA and TGF-β_1_. In addition, increased IL-6 production by M-MΦ as a result of the presence of profibrotic MyoFb cannot be excluded. Our observation of increased levels by TGF-β_1_ activation supports this fact, too. Thus, our results indicate feedback communication between MyoFb and M-MΦ, which produces an anti-fibrotic milieu, as recently discussed for alternatively activated, i.e., pro-healing MΦ by MyoFb-derived IL-6 [45]. Paracrine communication via IL-6 is, thus, a homeostatic regulatory pathway in wound healing that may also limit undesirable fibrosis. However, we also observed in our setting that matrix-bound sulfated GAGs do not modulate this cell–cell interaction as we did not observe any dependence from the heparin modification of the Coll I matrices. For IL-6, this fits expectations, as the weakly charged cytokine is known to bind sulfated GAGs only with low affinity, as discussed for IL-10, above [31,37].

Finally, we analyzed FGF-2, which modulates the growth, differentiation, and migration of a wide variety of cell types and is present throughout the wound healing process [46]. Furthermore, sulfated GAGs, like heparin and heparan sulfate (HS), are known to be essential for the biological activity of FGF-2 in vivo [47,48]. Those GAGs function as low-affinity receptors that bind, dimerize, and present FGF-2 to the FGF receptor. This binding is driven by the high positive charge of FGF-2 (IEP 9.6) [49]. In our experiments, the measured amount of FGF-2 in the supernatant of M-MΦ was approximately 25 pg mL^−1^ for all samples, independent of TGF-β_1_ application and network modification (Figure 2D). MyoFb produced slightly higher levels of FGF-2, however, with a twofold increase when matrices were modified with heparin. The same pattern was also observed in the coculture setting of MyoFb and M-MΦ. No dependence on TGF-β_1_ activation was observed for all samples. Our results clearly show that heparin but not 6-ON-desulfated heparin increases the concentration of FGF-2 in the supernatant, suggesting an autocrine regulation mediated by the matrix-bound sulfated GAG; however, this is dependent on the degree of sulfation. Hence, matrix-modulated autocrine FGF-2 signaling is suggested to occur in our biomimetic coculture setup of a late wound healing phase. This finding is in line with in vivo and in vitro reports that FGF-2 critically contributes to the wound healing process. The local administration of FGF-2 has been shown to have an anti-fibrotic effect on MyoFb through its inhibitory effect on their contractility [50] as well as the production of αSMA [51] and fibronectin [52]. There is also evidence that FGF-2 can accelerate wound closure in combination with TGF-β_1_ [53]. FGF-2 levels found in our experiments are much lower than for IL-8 and IL-6. This fact fits the idea that the matrix modulation of cell–cell signaling sensitively depends not only on the affinity of the specific cytokine to the GAG modification of the matrix but, further, on the relative amount of sulfated GAG in the matrix.

In sum, the multiplex ELISA analysis of relevant cytokines in late-stage wound healing shows that cell–cell communication of MyoFb and M-MΦ in a coculture can be partially modulated by functionalization of the matrix with sulfated GAGs. In addition to the necessary affinity of cytokines to sulfated GAGs, the amount of GAG-related binding sites in the matrix in relation to the amount of secreted GAGs is essential to achieve a matrix modulation of cytokine-related signaling. While the latter is apparent for the autocrine acting FGF-2 produced by MyoFb, the very high levels of M-MΦ-produced IL-8 override any matrix modulation. Furthermore, stimulating and synchronizing paracrine IL-6 signaling in the coculture setting was shown, which were additionally controlled by TGF-β_1_ activation. However, in this case, the low affinity of IL-6 to sulfated GAGs, and high cytokine levels, did not result in a matrix modulation, which can be similarly stated for IL-10.

## 3. Conclusions

**Figure 3:**
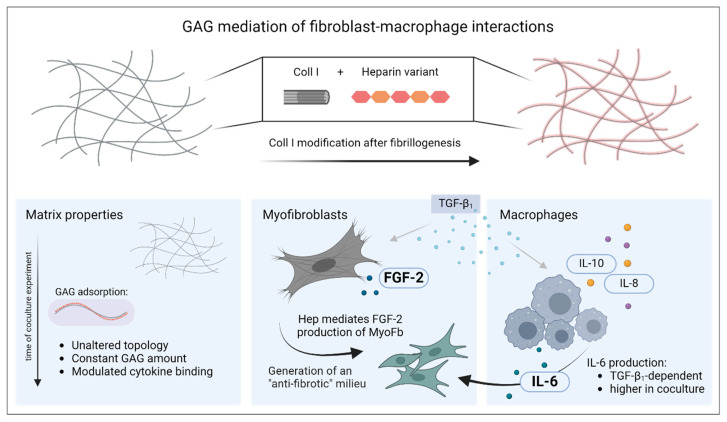
Mediation of fibroblast–macrophage interactions by functionalization of 3D collagen I networks with sulfated glycosaminoglycans. For biomimetic 3D scaffold preparation, modification of Coll I fibrils with GAG variants was performed after fibrillogenesis. The figure describes the changes of the three main components of the culture system over the period of 7 d: Coll I matrix, myofibroblasts and macrophages. The figure was generated using biorender.com (accessed on 11 June 2024).

In this study, we successfully introduced a biomimetic model of late-stage wound healing to modify interaction of different cell types (here, MyoFb and M-MΦ) in coculture using the adsorptive binding of sulfated GAGs to 3D fibrillar Coll I networks (see Figure 3). The functionalization of the Coll I scaffold with GAGs was performed after the formation of fibrillar hydrogels and was stable over several days. Moreover, the viability of the cells was not affected during scaffold reconstitution. Cytokine levels assessed by multiplex ELISA showed characteristic differences induced by paracrine or autocrine action and GAG functionalization of the Coll I-based networks, like a synchronized mode of action in cell–cell communication for IL-6 and heparin-regulated activation of autocrine FGF-2 signals.

In sum, we created a promising 3D ECM model with heparin or 6-ON-desulfated heparin modifications for further studies investigating the regulation of MyoFb by M-MΦ through soluble mediators. In addition to the shown influence on autocrine and paracrine acting meditators, the sulfated GAG modification of the 3D Coll I networks can also be applied to study the loading and release of heparin-binding drugs and active small molecules to the scaffolds and their influences on cell behavior in wound healing studies and other coculture systems. Future studies should also try to validate the usage of such in vitro scaffolds as a replacement for animal wound healing studies in comparison to in vivo experiments.

## 4. Materials and Methods

In the following, all materials and methods are described in brief only. For details, please refer to the Supplementary Materials.

### 4.1. Isolation and Culture of Primary Human Macrophages and Fibroblasts

Cell isolation and subsequent cell experiments were carried out in accordance with the approved guidelines of the ethics committee of the Medical Faculty Leipzig including written informed consent from healthy donors (ethics committee vote: 384/16-ek).

Human peripheral blood mononuclear cells were obtained from buffy coats from healthy donors. Monocytes were subsequently isolated using the counter-flow elutriation method and suspended at a concentration of 5 × 10^5^ mL^−1^ in M-MΦ cultivation medium (RPMI 1640 medium; Biochrom KG, Berlin, Germany) containing 10% *v*/*v* heat-inactivated fetal calf serum (FCS; Sigma-Aldrich, St. Louis, MO, USA), 100 U mL^−1^ penicillin (Merck, Darmstadt, Germany), 100 mg mL^−1^ streptomycin (Merck), and 50 ng mL^−1^ M-CSF (Life Technologies, Darmstadt, Germany) to differentiate towards M-MΦ at 37 °C, 95% humidity, and 5% CO_2_ in low-adherence polymer bags (fluorinated ethylene propylene (FEP) foil, 50 μm, hydrophobic; Zell-Kontakt, Nörte-Hardenberg, Germany) for 7 days.

Primary human dermal Fb were obtained and isolated from foreskin and subsequently expanded up to 6th passage. Prior to coculture experiments, Fb were adapted to M-MΦ cultivation medium (except M-CSF), and differentiation into MyoFb was undertaken by stimulation with recombinant human TGF-β_1_ (10 ng mL^−1^; Peprotech, Hamburg, Germany) for 2 days. Cell experiments were performed for 4 or 7 days either with or without further TGF-β_1_ (10 ng mL^−1^) treatment.

### 4.2. Cell Seeding during Reconstitution of 3D Coll I Matrices

Reconstitution of 3D Coll I matrices (from rat tail Coll I stock solution, Corning, New York, NY, USA) was performed on glass coverslips (diameter 13 mm or 20 mm) coated with poly(styrene-*alt*-maleic anhydride) (PSMA; MW 30,000 g mol^−1^) (Sigma-Aldrich). Corresponding numbers of M-MΦ and Fb were resuspended in the necessary amount of 250 mM phosphate buffer (Sigma-Aldrich), with pH adjusted to 7.5 by NaOH (Grüssing, Filsum, Germany). Coll I fibril formation was immediately initiated by transfer to 37 °C (95% relative humidity, 5% CO_2_) for at least 50 min in a wet chamber. After completing Coll I fibril formation, Coll I matrices were washed three times with PBS and immediately covered with 1 mL M-MΦ cultivation medium.

### 4.3. Kinetic Analysis of Coll I Fibril Formation

To monitor Coll I fibril formation in the presence of cells, Coll I cell solutions were prepared as described above and were analyzed in 96-well plates in a preheated plate reader at 37 °C (Tecan Infinite F200 Pro, Tecan, Männedorf, Switzerland). Turbidity was measured at 405 nm for 90 min at 1 min intervals.

### 4.4. Heparin Modification of Coll I Matrices and Quantification of Heparin Amount

After reconstitution, matrices were washed twice with PBS and 300 µL of a 0.1 mg mL^−1^ heparin (Sigma-Aldrich), or 6-ON-desulfated heparin solution (synthesized and characterized as described by [54]; dissolved in PBS) was added for 30 and 60 min at cell culture conditions with subsequent washing and adding of cultivation medium.

Quantification of heparin amount in 3D Coll I matrices was performed on day 1 and day 4 using atto550-fluorescently labelled heparin derivatives, digestion of the Coll I matrices by papain solution, and fluorescence measurement of digested matrix in a black 96-well microtiter plate at 535 nm/590 nm.

### 4.5. Characterization of Coll I Matrix Topology

Coll I matrix topology regarding pore and fibril diameter was characterized after fixation with 4% paraformaldehyde (PFA) for 15 min and subsequent staining with TAMRA-SE using a confocal laser scanning microscope 700 (cLSM 700, Zeiss, Jena, Germany) and a home-built image analysis tool.

### 4.6. WST-1 Assay

Cellular viability after incorporation into Coll I matrices and heparin modification was evaluated using WST-1 assay (Cayman Chemical, Ann Arbor, MI, USA) in 24-well plates and subsequent absorption measurements in a 96-well plate at 450 nm in a plate reader (Tecan Infinite F200 Pro, Männedorf, Switzerland).

### 4.7. Cytokine Analysis

Released cytokine concentrations were determined in supernatants after 7 days of cell culture using a multiplex immunoassay (ProcartaPlex^TM^, ThermoFisher Scientific, Waltham, MA, USA) according to manufacturer’s instructions.

### 4.8. Statistical Analysis

Unless otherwise indicated, all experiments were performed at least three times (*n* = 3), and data are presented as arithmetic means; error bars represent the standard deviation of the mean. Statistical analysis was performed using GraphPad Prism 6 software (GraphPad Software, Inc., La Jolla, CA, USA). One-way ANOVA was used to compare samples from the cell viability data. For the analysis of quantification of heparin amount and ELISA data, Tukey’s multiple comparison test followed by two-way ANOVA was performed. The significance level was set at *p* < 0.05 (*), while *p* < 0.01 (**) and *p* < 0.001 (***) were considered very and highly significant, respectively. Unmarked groups did not show significant differences.

## Data Availability

The Matlab script for network topology analysis is freely available at https://git.sc.uni-leipzig.de/pe695hoje/topology-analysis (accessed on 11 June 2024).

## Supplementary Materials

The following supporting information can be downloaded at: https://www.mdpi.com/article/10.3390/gels10070450/s1, Supporting Information including Supplementary Figures S1–S3 with experimental details (DOC). Figure S1: Analysis of GAG amount in Coll I matrices without incorporated cells 1 d and 4 d after reconstitution using papain digestion. Figure S2: Detection of cell viability after 4 d of cell culture shown for heparin-modified Coll I matrices after 15, 30 and 60 min of GAG incubation. Figure S3: Topology analysis of Coll I matrices from MyoFb-M-MΦ coculture. References [20,40,41,54,55,56,57,58,59] are cited in the supplementary materials.
