# Supplementary material for: The Influence of Sulfation Degree of Glycosaminoglycan-Functionalized 3D Collagen I Networks on Cytokine Profiles of In Vitro Macrophage–Fibroblast Cocultures
Source: Gels. 2024 Jul 9;10(7):450. doi: 10.3390/gels10070450 (PMC11276094; doi:10.3390/gels10070450)
Supplement: Supplementary file 1 [file gels-10-00450-s001.zip › gels-3078643-supplementary.pdf]

Supplementary Materials

The Influence of Sulfation Degree of Glycosaminoglycan Functionalized 3D Collagen I Networks on Cytokine Profiles of *In Vitro* Macrophage—Fibroblast Cocultures

Franziska Ullm[1], Alexander Renner[1], Uwe Freudenberg[2], Carsten Werner[2], Tilo Pompe[1,2]

[1] Leipzig University, Institute of Biochemistry, Johannisallee 21-23, 04103 Leipzig, Germany

[2] Leibniz-Institut für Polymerforschung Dresden e.V., Max Bergmann Center of Biomaterials, Hohe Strasse 6, 01069 Dresden, Germany

1. Supplementary Materials and Methods

1.1. Isolation and Culture of Primary Human Macrophages and Fibroblasts

Cell isolation and subsequent cell experiments were carried out in accordance with the approved guidelines of the ethics committee of the Medical Faculty Leipzig including written informed consent from healthy donors (ethics committee vote: 384/16-ek).

Buffy coats from healthy donors were acquired from the Institute of Transfusion Medicine, University Hospital Leipzig. The buffy coats were diluted 1:1 with sterile PBS and human peripheral blood mononuclear cells were obtained using Ficoll-Paque Plus centrifugation (GE Healthcare, Uppsala, Sweden), as described in detail elsewhere [55]. Cells were washed three times in PBS containing 0.3 mM EDTA (Sigma-Aldrich, Steinheim, Germany) before proceeding to monocyte isolation using the counter-flow elutriation method applying the JE-5.0 elutriation system (Beckman Coulter, Brea, CA), as described in detail elsewhere [56]. Monocytes were suspended at a concentration of $5 \cdot 10^5$ mL^{-1} in M-MΦ cultivation medium (RPMI 1640 medium; Biochrom KG, Berlin, Germany) containing 10% v/v heat inactivated fetal calf serum (FCS; Sigma-Aldrich), 100 U mL^{-1} penicillin (Merck, Darmstadt, Germany), 100 mg mL^{-1} streptomycin (Merck), and 50 ng mL^{-1} M-CSF (Life Technologies, Darmstadt, Germany). They were differentiated towards M-MΦ at 37 °C, 95% humidity, and 5% CO_2 in low-adherence polymer bags (fluorinated ethylene propylene (FEP) foil, 50 µm, hydrophobic; Zell-Kontakt, Nörte-Hardenberg, Germany) for 7 days (see details elsewhere) [57]. After the differentiation period, M-MΦ were harvested from the FEP bags and again suspended in M-MΦ cultivation medium at a concentration of $1 \cdot 10^6$ mL^{-1} prior use.

Primary human dermal Fb were obtained from foreskin according to an isolation protocol described by Saalbach et al. [58]. Cells were expanded up to 6th passage. For adaptation to *in vitro* cell culture conditions, initial cell culture was performed with Dulbecco's modified Eagle's medium (DMEM) (Biochrom) supplemented with 10% v/v FCS (Sigma-Aldrich) and 1% v/v ZellShield (antibiotics) (Biochrom). Prior to coculture experiments, FB were adapted to M-MΦ cultivation medium (except M-CSF) for 5 days. The differentiation of Fb into MyoFb was undertaken by stimulation with recombinant human TGF-β_1 (10 ng mL^{-1}; Peprotech, Hamburg, Germany) 2 days before coculture experiments started. Successful differentiation to myofibroblasts has already been demonstrated using this protocol [59]. Cell experiments were performed for 4 or 7 days either with or without further TGF-β_1 (10 ng mL^{-1}) treatment.

1.2. Cell Seeding During Reconstitution of 3D Coll I Matrices

Reconstitution of 3D Coll I matrices was done on glass coverslips (diameter 13 mm or 20 mm) coated with poly(styrene-*alt*-maleic anhydride) (PSMA; MW 30 000 g mol^{-1}) (Sigma-Aldrich). Preparation procedure of glass coverslips and 3D Coll I matrices is described in detail elsewhere [60] [61]. Coll I preparation prior to matrix reconstitution was performed on ice. In brief, Coll I stock solution (rat tail, Corning, New York) was prediluted in acetic acid (0.02 N). After counting and centrifugation, corresponding numbers of M-MΦ and Fb were resuspended in the necessary amount of 250 mM phosphate buffer (Sigma-Aldrich), pH adjusted to 7.5 by NaOH (Grüssing, Filsum, Germany) supplement). In order to produce the Coll I reconstitution solution, cell containing phosphate buffer and prediluted Coll I stock solution were thoroughly mixed (final concentrations: Coll I 2.5 mg mL^{-1}, M-MΦ: $2 \cdot 10^6$ mL^{-1}, Fb: $0.5 \cdot 10^6$ mL^{-1} (if not stated otherwise). For 13 mm cover slips, a total volume of 40 µL of the Coll I-cell reconstitution solution were used which was adapted to 90 µL for preparing networks on 20 mm cover slips. Coll I fibril formation was immediately initiated by transfer to 37 °C (95% relative humidity, 5% CO_2) for at least 50 min in a wet chamber. After completing Coll I fibril formation, Coll I matrices were washed three times with PBS and immediately covered with 1 mL M-MΦ cultivation medium.

1.3. Kinetic Analysis of Coll I Fibril Formation

To monitor Coll I fibril formation in the presence of cells, Coll I - cell solutions were prepared as described above. 100 µL of the solutions were transferred to a precooled 96-well plate and placed immediately in a preheated plate reader at 37 °C (Tecan Infinite F200 Pro, Tecan, Männedorf, Switzerland). Turbidity was measured at 405 nm for 90 min at 1 min intervals. Measurements were performed in technical and biological triplicates (n=3). Turbidity-time curves were averaged for every condition.

1.4. Heparin Modification of Coll I Matrices and Quantification of Heparin Amount

Between completed fibrillation of Coll I and heparin modification, incorporated cells were allowed to regenerate under standard cell culture conditions for at least 1 h. Afterwards, matrices were washed twice with PBS and 300 µL of a 0.1 mg mL^{-1} heparin (Sigma-Aldrich) or 6-ON-desulfated heparin solution (synthesized and characterized as described by [62]; dissolved in PBS) was added. 3D Coll I matrices were incubated under standard cell culture conditions for 30 and 60 min. After incubation, heparin solution was removed and 3D Coll I

matrices were washed three times with PBS, 5 min each. Subsequently, cultivation medium was added.

Quantification of heparin amount in 3D Coll I matrices was done at day 1 and day 4 after modification using atto550-fluorescently labelled heparin derivatives (synthesized and characterized as described by [62]. To first digest the Coll I matrices, papain solution was prepared using 0.6 mg mL^{-1} L-cysteine and 0.95 µL mL^{-1} papain from papaya latex in a 0.01 M solution of EDTA in PBS. Next, 300 µL of papain solution were added to each well and the matrices were digested for 2 h at 60 °C. After 1 h of incubation, the plates were manually stirred by shaking. Fluorescence of digested matrix solutions was measured in a black 96 well micro titre plate in technical duplicates at 535 nm / 590 nm. The concentrations of atto550-heparin and atto550-6-ON-desulfated heparin adsorbed to the matrices were determined from the intensities of fluorescence via calibrating curves. The experiment was independently conducted 3 times.

1.5. Characterization of Coll I Matrix Topology

Coll I matrix topology regarding pore and fibril diameter was characterized using a home-built image analysis tool, as described previously [63] [61]. After culturing, Coll I matrices were washed with PBS three times and cells incorporated were fixated with 4% paraformaldehyde (PFA) for 15 min. Afterwards, matrices were rinsed with PBS three times. Further, Coll I matrices were incubated with 400 µL of a TAMRA-SE solution (in PBS, 50 µM) for 60 min at room temperature to visualize the microstructure of the Coll I matrices. Subsequently, Coll I matrices were again rinsed three times with PBS for 5 min each and then imaged using a confocal laser scanning microscope 700 (cLSM 700, Zeiss, Jena, Germany) as described earlier [63]. Experiments were performed in independent triplicates.

1.6. WST-1 Assay

Cellular viability after incorporation into Coll I matrices and heparin modification were evaluated using WST-1 assay. For preparation, cell-containing Coll I matrices were washed twice with PBS. Cell proliferation reagent WST-1 and the electron coupling solution (Cayman Chemical, Ann Arbor, MI) were mixed in a ratio of 1:1. After a 1:10 dilution in phenol red free RPMI-1640 medium (Biochrom), 300 µL of the solution were applied to each well. The 24 well plates were then incubated at 37 °C for 1 h. After incubation, 75 µL of each well were transferred into a 96 well plate and the absorption was measured at 450 nm in triplicates in a

plate reader. Obtained values were then normalized to unmodified samples, so a baseline of 1 corresponds to an equivalent viability as in the control. The assay was conducted three times in independent cell experiments.

1.7. Cytokine Analysis

Released cytokine concentrations were determined in supernatants after 7 days of cell culture using a multiplex immunoassay (ProcartaPlex™, ThermoFisher Scientific, Waltham, MA) according to manufacturer's instructions. 1 mL of cell culture supernatants were collected on ice and centrifuged with 362 *g* for 10 min at 4 °C to remove cell debris. Supernatants were stored at -20 °C until analysis. The assay was performed with the supernatants of three independent cell experiments.

1.8. Statistical analysis

Unless otherwise indicated, all experiments were performed at least three times ($n = 3$), and data are presented as arithmetic means; error bars represent the standard deviation of the mean. Statistical analysis was performed using GraphPad Prism6 software (GraphPad Software, Inc., CA). One-way ANOVA was used to compare samples from the cell viability data. For the analysis of quantification of heparin amount and ELISA data, Tukey's multiple comparison test followed by two-way ANOVA was performed. The significance level was set at $p < 0.05$ (*), while $p < 0.01$ (**) and $p < 0.001$ (***) were considered very and highly significant, respectively. Unmarked groups did not show significant differences.

2. Supplementary Figures

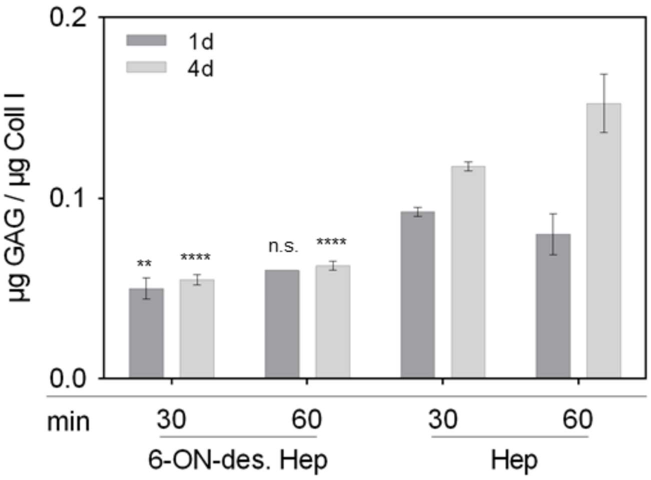

Figure S1: Analysis of GAG amount in Coll I matrices without incorporated cells 1 d and 4 d after reconstitution using papain digestion (n = 4; data is given as mean ± SE; Tukey's multiple comparisons test followed by two-way ANOVA; * indicates $p \leq 0.05$; ** indicates $p \leq 0.005$; *** indicates $p \leq 0.001$; **** indicates $p \leq 0.0001$ between the respective heparin variants).

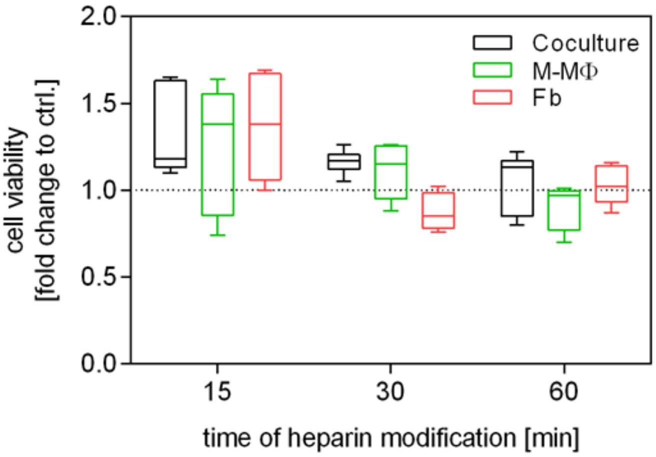

Figure S2: Detection of cell viability after 4 d of cell culture shown for heparin-modified Coll I matrices after 15, 30 and 60 min of GAG incubation (n = 3, Box-whisker plots show the 25 and 75 percentile range (box) with Tukey 95% confidence intervals (whiskers) and median values (transversal line); dotted line represents respective Coll I control without GAG modification).

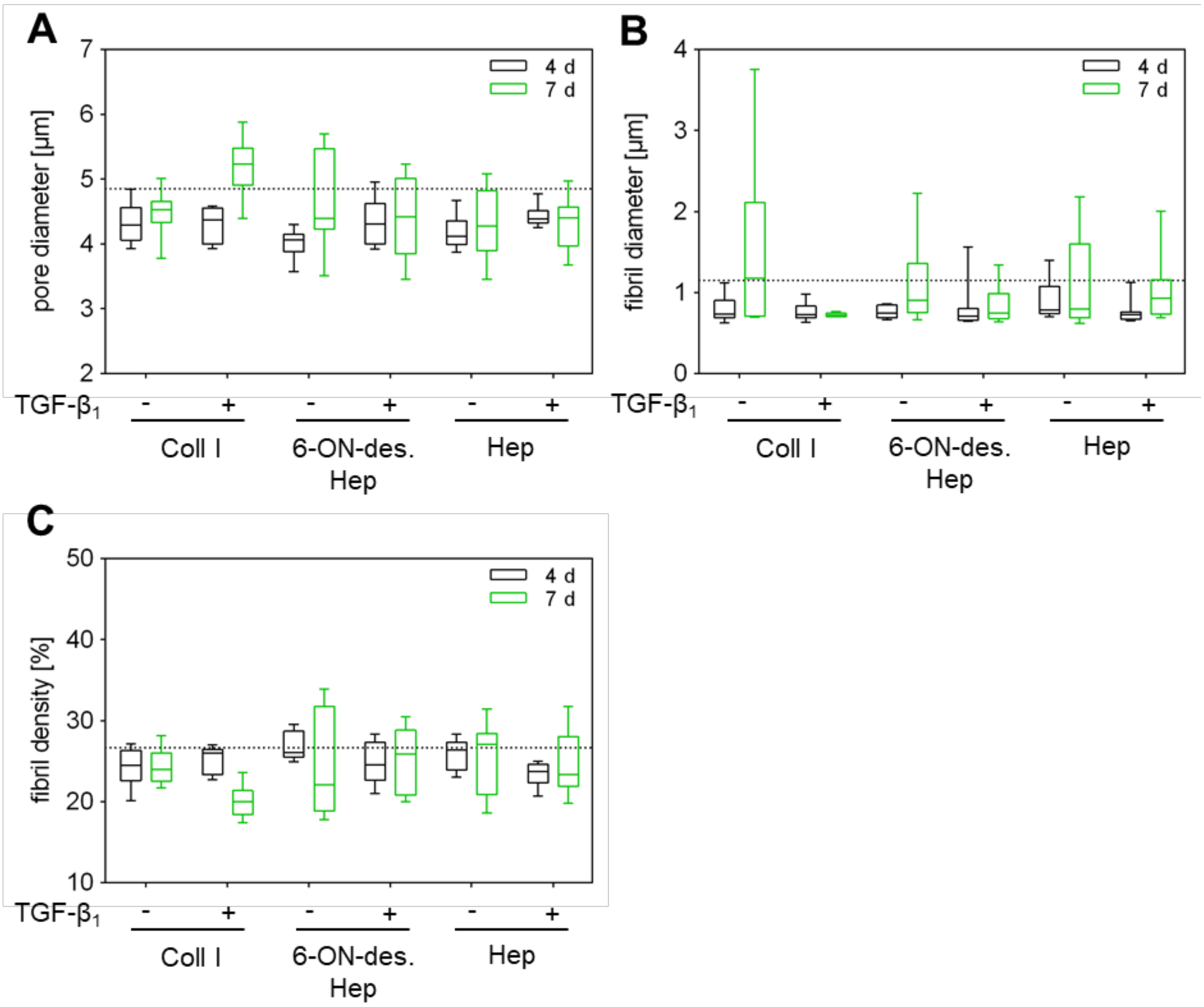

Figure S3: Topology analysis of Coll I matrices from MyoFb-M-MΦ coculture. **A)** Pore diameter, **B)** fibril diameter and **C)** fibril density were examined after 4 d and 7 d of coculture (n = 3, Box-whisker plots show the 25 and 75 percentile range (box) with Tukey 95% confidence intervals (whiskers) and median values (transversal line)). Dotted line represents values of respective 2.5 mg mL^{-1} Coll I control without incorporated cells.
